# Polymyxin-Resistant *Acinetobacter* spp. Isolates: What is Next?

**DOI:** 10.3201/eid0908.030052

**Published:** 2003-08

**Authors:** Adriana O. Reis, Deise A.M. Luz, Maria C.B. Tognim, Hélio S. Sader, Ana C. Gales

**Affiliations:** * Universidade Federal de São Paulo, São Paulo, Brazil

**Keywords:** *Acinetobacter* spp., polymyxins, molecular epidemiology, letter

**To the Editor**: In Brazilian hospitals, *Acinetobacter* spp. has been an important etiologic agent of nosocomial infections, mainly pneumonia (*1*–*3*). In general, ampicillin/sulbactam and carbapenems remain the last therapeutic options for treatment of such infections (*3*,*4*). However, resistance rates to carbapenems have increased, reaching rates approximately 12% or higher in some Brazilian hospitals (*1*,*3*,*4*). Thus, more toxic agents such as polymyxins have been used as alternative therapeutic drugs against multidrug-resistant *Acinetobacter* infections (*5*,*6*). The clinical use of polymyxins has been based on antimicrobial susceptibility results and previous clinical experience. However, the National Committee for Clinical Laboratory Standards (NCCLS) documents do not currently provide interpretative criteria for the testing of polymyxins (*7*). In addition, the disk diffusion technique was reported to be an unreliable method for evaluating the susceptibility to polymyxins (*8*). Since *Acinetobacter* clinical specimens exhibiting high MICs for polymyxins (MIC, 8–32 μg/mL) were recently detected, we searched for the frequency of occurrence of *Acinetobacter* spp. strains exhibiting reduced susceptibility to polymyxin B among 100 bloodstream isolates of *Acinetobacter* spp (*8*). The bacterial isolates were consecutively collected between September 1999 and December 2000 from a tertiary Brazilian hospital, where *Acinetobacter* spp. infections have reached endemic levels and polymyxins have been frequently used. Only one isolate per patient was included in the study.

The isolates were identified to the species level using the BBL Crystal System (Becton Dickinson, Sparks, MD). The susceptibility to polymyxin B and meropenem were tested by disk diffusion and agar dilution techniques according to NCCLS recommendations (*9*,*10*). The susceptibility interpretative criteria for meropenem and polymyxin B were based on the current and former NCCLS documents, respectively (*7*,*11*). The MIC was defined as the lowest antimicrobial concentration that inhibited bacterial growth. *Pseudomonas aeruginosa* ATCC 27853, *Staphylococcus aureus* ATCC 25923, and *Escherichia coli* ATCC 25922 were used as quality control strains. Testing errors and agreements were determined by comparing the results of the disk diffusion with the standard criterion agar dilution method. Categorical agreement was obtained when the isolates were classified within the same susceptibility category. The very major and major errors were related to false susceptibility and false resistance results, respectively. To evaluate whether the polymyxin B-resistant strains isolates were epidemiologically related, these isolates were molecularly typed by pulsed-field gel electrophoresis (PFGE) as previously described (*12*). PFGE patterns were considered identical if they shared every band, similar if they differed from one to three bands, and distinct if they differed by four or more bands (*12*).

Despite the limitation of commercial systems for identifying the genus *Acinetobacter* at species level, *Acinetobacter* *baumannii* (80.0%) was the most commonly identified species, followed by *A.* *lwoffi* (4.0%). Sixteen percent of the *Acinetobacter* isolates were not identified to species level by the BBL Crystal System. Meropenem (MIC50, 1 μg/mL) and polymyxin B (MIC50, 1 μg/mL) showed similar in vitro potency. However, meropenem exhibited the highest susceptibility rate (99.0% susceptible). In contrast to previous studies, only one strain was resistant to meropenem (*1*–*3*,*8*), which indicates that the carbapenem-susceptibility rates among *Acinetobacter* spp. isolates may vary according to the period evaluated even in the same institution. By using the polymyxin B resistance breakpoint (MIC >4 μg/mL) presented by the former NCCLS document, which was recently validated, we found that five *Acinetobacter* spp. isolates were considered resistant to polymyxin B (MICs, 8–32 μg/mL) (*8*,*11*). All isolates were susceptible to meropenem and belonged to *A. baumannnii* (*4*) and *A.* *lwoffi* (*1*) species. The polymyxin B–resistant isolates were categorized as susceptible by disk diffusion (100%, very major error). The disk diffusion method is widely used in Brazil and worldwide. However, disk diffusion was confirmed to be an unreliable test for detecting *Acinetobacter* spp. isolates with reduced susceptibility to polymyxins. These results are in agreement with those previously reported (*8*).

Among the five polymyxin B–resistant *Acinetobacter* spp., four distinct patterns were characterized by PFGE. Two polymyxin B–resistant strains, which were isolated from different units of the São Paulo Hospital complex, shared an identical PFGE pattern. The PFGE results suggest that the polymyxin B use may have played a role in the selection of resistant strains. On the other hand, two isolates shared an identical PFGE pattern, which raises the possibility of patient-to-patient transmission of epidemic strains. Intra- and interhospital dissemination of multidrug-resistant *Acinetobacter* spp. clones has already been reported in Brazilian hospitals (*13*).

Our findings suggest that the polymyxin B–resistant strains have emerged because of antimicrobial selective pressure and dissemination of clonal strains. Further epidemiologic studies are necessary to correlate the emergence of polymyxin-resistant *Acinetobacter* spp. isolates to the clinical response with polymyxin B therapy. Since the emergence of polymyxin B resistance may leave no efficacious drugs for the treatment of infections caused by multidrug-resistant *Acinetobacter* spp. isolates, strict infection control measures must be adopted to avoid the emergence and spread of such isolates. The low accuracy of routine susceptibility tests, especially disk diffusion, may jeopardize rapid implementation of such measures.

## References

[1] Sader HS, Gales AC, Pfaller MA, Mendes RE, Zoccoli C, Barth A, Pathogen frequency and resistance patterns in Brazilian hospitals: summary of results from three years of the SENTRY Antimicrobial Surveillance Program. Braz J Infect Dis. 2001;5:200–14. 10.1590/S1413-86702001000400006 11712965

[2] Gales AC, Jones RN, Forward KR, Linares J, Sader HS, Verhoef J. Emerging importance of multidrug-resistant *Acinetobacter* species and *Stenotrophomonas maltophilia* as pathogens in seriously ill patients: geographic patterns, epidemiological features, and trends in the SENTRY Antimicrobial Surveillance Program (1997–1999). Clin Infect Dis. 2001;32(Suppl 2):S104–13. 10.1086/320183 11320451

[3] Levin AS, Mendes CM, Sinto SI, Sader HS, Scarpitta CR, Rodrigues E, An outbreak of multiresistant *Acinetobacter baumanii* in a university hospital in São Paulo, Brazil. Infect Control Hosp Epidemiol. 1996;17:366–8. 10.1086/647319 8805068

[4] Gales AC, Sader HS, Sinto S, Santos OP, Mendes CMF. In vitro activity of ampicillin-sulbactam against clinical multiresistant *Acinetobacter baumannii* isolates. J Chemother. 1996;8:416–9. 8981180 10.1179/joc.1996.8.6.416

[5] Go ES, Urban C, Burns J, Kreiswirth B, Eisner W, Mariano N, Clinical and molecular epidemiology of *Acinetobacter* infections sensitive only to polymyxin B and sulbactam. Lancet. 1994;344:1329–32. 10.1016/S0140-6736(94)90694-7 7968028

[6] Levin AS, Barone AA, Penco J, Santos MV, Marinho IS, Arruda EA, Intravenous colistin as therapy for nosocomial infections caused by multidrug-resistant *Pseudomonas aeruginosa* and *Acinetobacter baumannii.* Clin Infect Dis. 1999;28:1008–11. 10.1086/514732 10452626

[7] National Committee for Clinical Laboratory Standards (NCCLS). Performance standards for antimicrobial disk susceptibility testing. Document M100-S12. Wayne (PA): The Committee; 2002.

[8] Gales AC, Reis AO, Jones RN. Contemporary assessment of antimicrobial susceptibility testing methods for polimyxin B and colistin: review of available interpretative criteria and quality control guidelines. J Clin Microbiol. 2001;39:183–90. 10.1128/JCM.39.1.183-190.2001 11136768 PMC87699

[9] National Committee for Clinical Laboratory Standards (NCCLS). Methods for dilution antimicrobial susceptibility test for bacteria that grow aerobically. 5th ed. Approved standard M7-A5. Wayne (PA): The Committee; 2000.

[10] National Committee for Clinical Laboratory Standards (NCCLS). Performance standards for antimicrobial disk susceptibility tests. Approved standard M2-A7. Wayne (PA): The Committee; 2000.

[11] National Committee for Clinical Laboratory Standards (NCCLS). Performance standards for antimicrobic disk susceptibility tests. Approved standard M2-A2 S2. Wayne (PA): The Committee; 1981.

[12] Pfaller MA, Sader HS, Hollis RJ. Chromosomal restriction fragment analysis by pulsed-field gel electrophoresis. Isenberg HD, editor. Vol. 1. Clinical microbiology procedures handbook. Washington: ASM Press; 1992. p. 1–12.

[13] Sader HS, Mendes CF, Pignatari AC, Pfaller MA. Use of macrorestriction analysis to demonstrate interhospital spread of multiresistant *Acinetobacter baumannii* in São Paulo, Brazil. Clin Infect Dis. 1996;23:631–4. 8879791 10.1093/clinids/23.3.631

